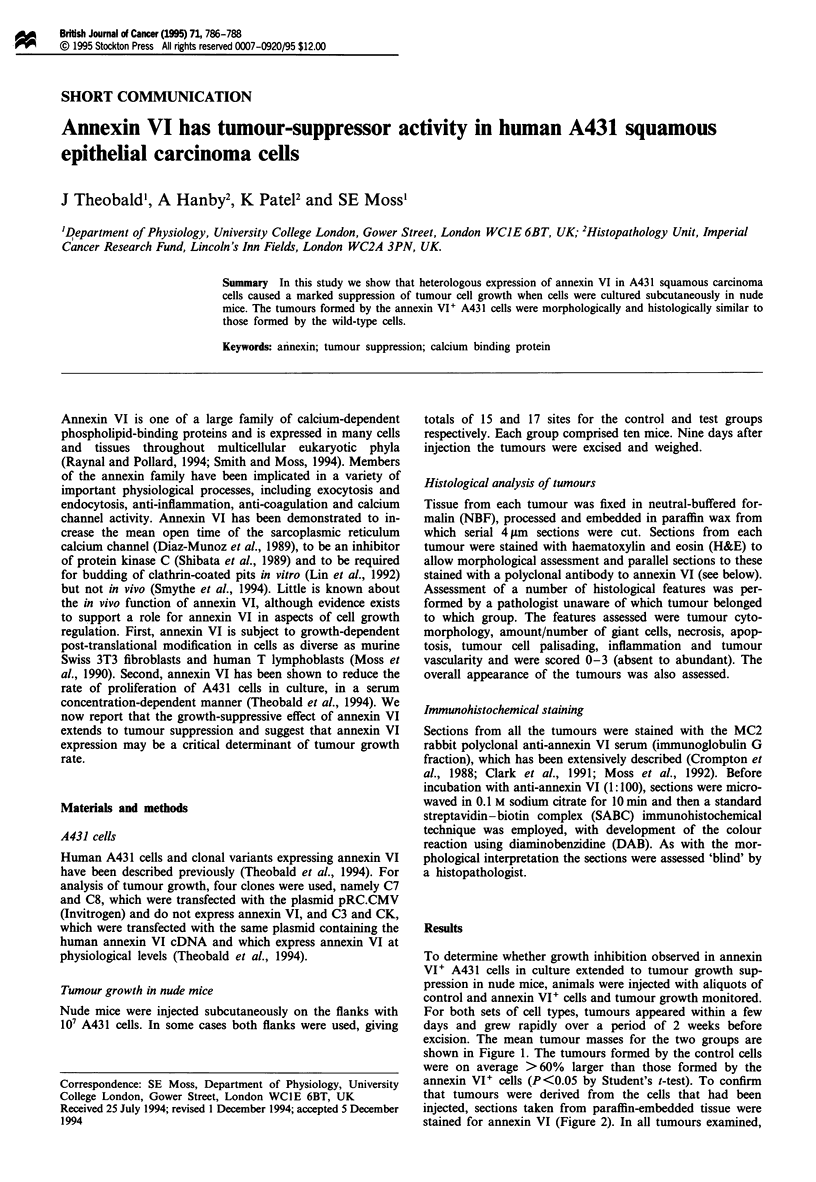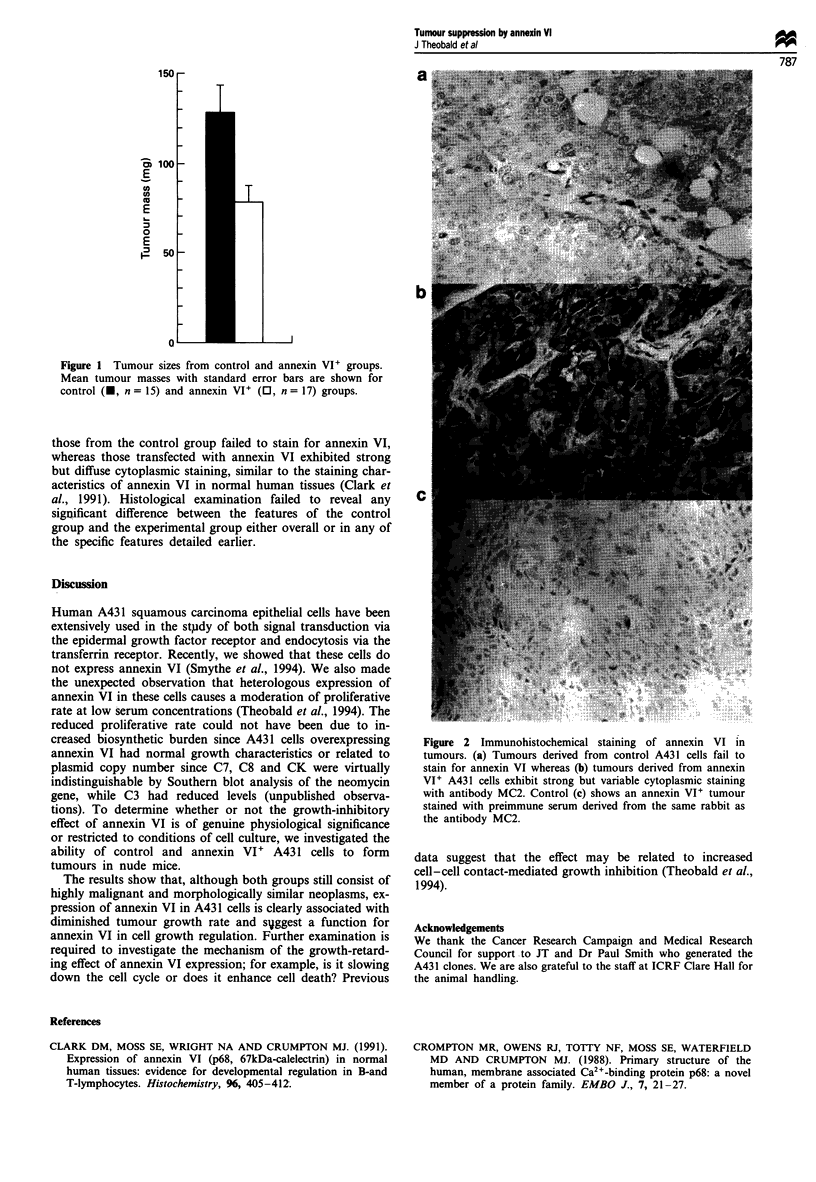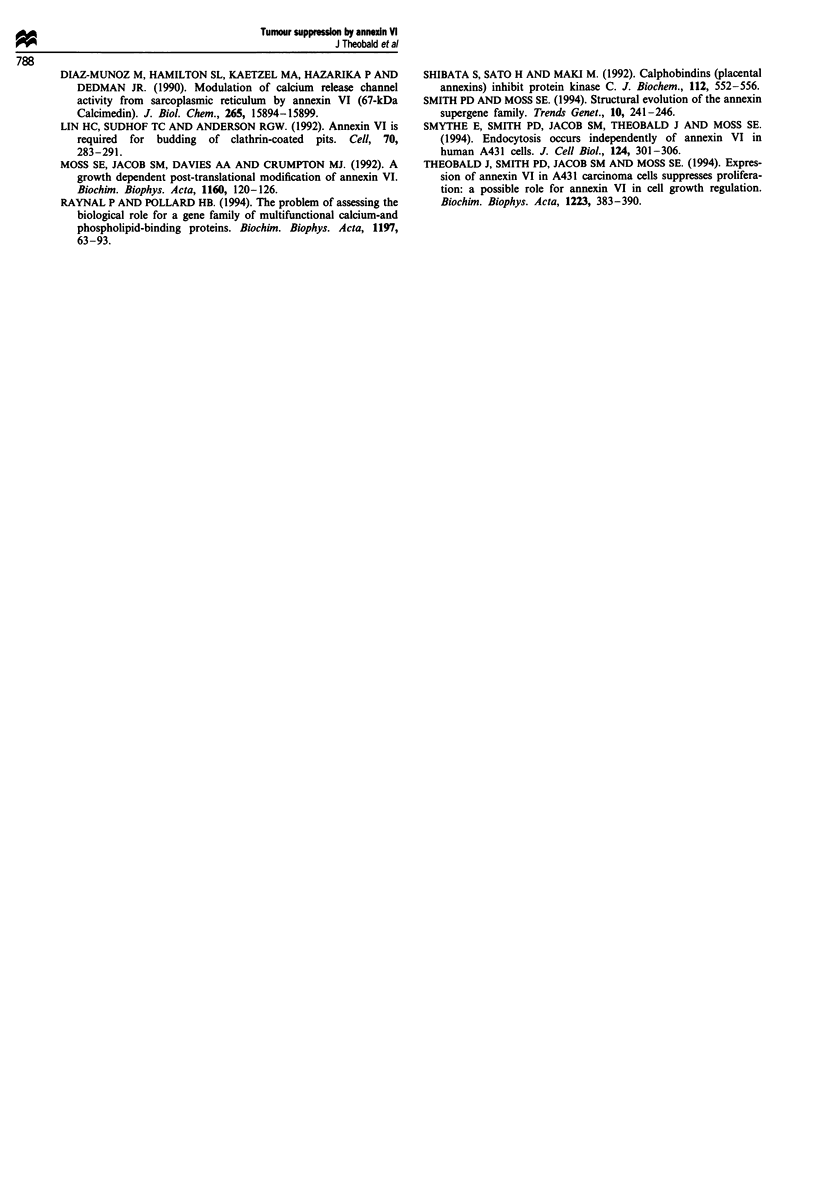# Annexin VI has tumour-suppressor activity in human A431 squamous epithelial carcinoma cells.

**DOI:** 10.1038/bjc.1995.152

**Published:** 1995-04

**Authors:** J. Theobald, A. Hanby, K. Patel, S. E. Moss

**Affiliations:** Department of Physiology, University College London, UK.

## Abstract

**Images:**


British Journal of Cancer (1995) 71, 786–788
© 1995 Stockton Press All rights reserved 0007-0920/95 $12.00

SHORT COMMUNICATION

### Annexin VI has tumour-suppressor activity in human A431 squamous epithelial carcinoma cells

J Theobald[1], A Hanby[2], K Patel[2] and SE Moss[1]

[1]*Department of Physiology, University College London, Gower Street, London WC1E 6BT, UK;* [2]*Histopathology Unit, Imperial Cancer Research Fund, Lincoln's Inn Fields, London WC2A 3PN, UK.*

**Summary** In this study we show that heterologous expression of annexin VI in A431 squamous carcinoma cells caused a marked suppression of tumour cell growth when cells were cultured subcutaneously in nude mice. The tumours formed by the annexin VI$^+$ A431 cells were morphologically and histologically similar to those formed by the wild-type cells.

**Keywords:** annexin; tumour suppression; calcium binding protein

Annexin VI is one of a large family of calcium-dependent phospholipid-binding proteins and is expressed in many cells and tissues throughout multicellular eukaryotic phyla (Raynal and Pollard, 1994; Smith and Moss, 1994). Members of the annexin family have been implicated in a variety of important physiological processes, including exocytosis and endocytosis, anti-inflammation, anti-coagulation and calcium channel activity. Annexin VI has been demonstrated to increase the mean open time of the sarcoplasmic reticulum calcium channel (Diaz-Munoz *et al.*, 1989), to be an inhibitor of protein kinase C (Shibata *et al.*, 1989) and to be required for budding of clathrin-coated pits *in vitro* (Lin *et al.*, 1992) but not *in vivo* (Smythe *et al.*, 1994). Little is known about the *in vivo* function of annexin VI, although evidence exists to support a role for annexin VI in aspects of cell growth regulation. First, annexin VI is subject to growth-dependent post-translational modification in cells as diverse as murine Swiss 3T3 fibroblasts and human T lymphoblasts (Moss *et al.*, 1990). Second, annexin VI has been shown to reduce the rate of proliferation of A431 cells in culture, in a serum concentration-dependent manner (Theobald *et al.*, 1994). We now report that the growth-suppressive effect of annexin VI extends to tumour suppression and suggest that annexin VI expression may be a critical determinant of tumour growth rate.

### Materials and methods

### *A431 cells*

Human A431 cells and clonal variants expressing annexin VI have been described previously (Theobald *et al.*, 1994). For analysis of tumour growth, four clones were used, namely C7 and C8, which were transfected with the plasmid pRC.CMV (Invitrogen) and do not express annexin VI, and C3 and CK, which were transfected with the same plasmid containing the human annexin VI cDNA and which express annexin VI at physiological levels (Theobald *et al.*, 1994).

### *Tumour growth in nude mice*

Nude mice were injected subcutaneously on the flanks with $10^7$ A431 cells. In some cases both flanks were used, giving totals of 15 and 17 sites for the control and test groups respectively. Each group comprised ten mice. Nine days after injection the tumours were excised and weighed.

### *Histological analysis of tumours*

Tissue from each tumour was fixed in neutral-buffered formalin (NBF), processed and embedded in paraffin wax from which serial 4 μm sections were cut. Sections from each tumour were stained with haematoxylin and eosin (H&E) to allow morphological assessment and parallel sections to these stained with a polyclonal antibody to annexin VI (see below). Assessment of a number of histological features was performed by a pathologist unaware of which tumour belonged to which group. The features assessed were tumour cytomorphology, amount/number of giant cells, necrosis, apoptosis, tumour cell palisading, inflammation and tumour vascularity and were scored 0–3 (absent to abundant). The overall appearance of the tumours was also assessed.

### *Immunohistochemical staining*

Sections from all the tumours were stained with the MC2 rabbit polyclonal anti-annexin VI serum (immunoglobulin G fraction), which has been extensively described (Crompton *et al.*, 1988; Clark *et al.*, 1991; Moss *et al.*, 1992). Before incubation with anti-annexin VI (1:100), sections were microwaved in 0.1 M sodium citrate for 10 min and then a standard streptavidin–biotin complex (SABC) immunohistochemical technique was employed, with development of the colour reaction using diaminobenzidine (DAB). As with the morphological interpretation the sections were assessed 'blind' by a histopathologist.

### Results

To determine whether growth inhibition observed in annexin VI$^+$ A431 cells in culture extended to tumour growth suppression in nude mice, animals were injected with aliquots of control and annexin VI$^+$ cells and tumour growth monitored. For both sets of cell types, tumours appeared within a few days and grew rapidly over a period of 2 weeks before excision. The mean tumour masses for the two groups are shown in Figure 1. The tumours formed by the control cells were on average >60% larger than those formed by the annexin VI$^+$ cells ($P<0.05$ by Student's $t$-test). To confirm that tumours were derived from the cells that had been injected, sections taken from paraffin-embedded tissue were stained for annexin VI (Figure 2). In all tumours examined,

Correspondence: SE Moss, Department of Physiology, University College London, Gower Street, London WC1E 6BT, UK
Received 25 July 1994; revised 1 December 1994; accepted 5 December 1994

**Figure 1** Tumour sizes from control and annexin VI$^+$ groups. Mean tumour masses with standard error bars are shown for control (■, $n = 15$) and annexin VI$^+$ (□, $n = 17$) groups.

those from the control group failed to stain for annexin VI, whereas those transfected with annexin VI exhibited strong but diffuse cytoplasmic staining, similar to the staining characteristics of annexin VI in normal human tissues (Clark *et al.*, 1991). Histological examination failed to reveal any significant difference between the features of the control group and the experimental group either overall or in any of the specific features detailed earlier.

### Discussion

Human A431 squamous carcinoma epithelial cells have been extensively used in the study of both signal transduction via the epidermal growth factor receptor and endocytosis via the transferrin receptor. Recently, we showed that these cells do not express annexin VI (Smythe *et al.*, 1994). We also made the unexpected observation that heterologous expression of annexin VI in these cells causes a moderation of proliferative rate at low serum concentrations (Theobald *et al.*, 1994). The reduced proliferative rate could not have been due to increased biosynthetic burden since A431 cells overexpressing annexin VI had normal growth characteristics or related to plasmid copy number since C7, C8 and CK were virtually indistinguishable by Southern blot analysis of the neomycin gene, while C3 had reduced levels (unpublished observations). To determine whether or not the growth-inhibitory effect of annexin VI is of genuine physiological significance or restricted to conditions of cell culture, we investigated the ability of control and annexin VI$^+$ A431 cells to form tumours in nude mice.

The results show that, although both groups still consist of highly malignant and morphologically similar neoplasms, expression of annexin VI in A431 cells is clearly associated with diminished tumour growth rate and suggest a function for annexin VI in cell growth regulation. Further examination is required to investigate the mechanism of the growth-retarding effect of annexin VI expression; for example, is it slowing down the cell cycle or does it enhance cell death? Previous data suggest that the effect may be related to increased cell–cell contact-mediated growth inhibition (Theobald *et al.*, 1994).

**Figure 2** Immunohistochemical staining of annexin VI in tumours. (**a**) Tumours derived from control A431 cells fail to stain for annexin VI whereas (**b**) tumours derived from annexin VI$^+$ A431 cells exhibit strong but variable cytoplasmic staining with antibody MC2. Control (**c**) shows an annexin VI$^+$ tumour stained with preimmune serum derived from the same rabbit as the antibody MC2.

**Acknowledgements**
We thank the Cancer Research Campaign and Medical Research Council for support to JT and Dr Paul Smith who generated the A431 clones. We are also grateful to the staff at ICRF Clare Hall for the animal handling.

### References

CLARK DM, MOSS SE, WRIGHT NA AND CRUMPTON MJ. (1991). Expression of annexin VI (p68, 67kDa-calelectrin) in normal human tissues: evidence for developmental regulation in B-and T-lymphocytes. *Histochemistry*, **96**, 405–412.

CROMPTON MR, OWENS RJ, TOTTY NF, MOSS SE, WATERFIELD MD AND CRUMPTON MJ. (1988). Primary structure of the human, membrane associated $Ca^{2+}$-binding protein p68: a novel member of a protein family. *EMBO J.*, **7**, 21–27.

DIAZ-MUNOZ M, HAMILTON SL, KAETZEL MA, HAZARIKA P AND DEDMAN JR. (1990). Modulation of calcium release channel activity from sarcoplasmic reticulum by annexin VI (67-kDa Calcimedin). *J. Biol. Chem.*, **265**, 15894–15899.

LIN HC, SUDHOF TC AND ANDERSON RGW. (1992). Annexin VI is required for budding of clathrin-coated pits. *Cell*, **70**, 283–291.

MOSS SE, JACOB SM, DAVIES AA AND CRUMPTON MJ. (1992). A growth dependent post-translational modification of annexin VI. *Biochim. Biophys. Acta*, **1160**, 120–126.

RAYNAL P AND POLLARD HB. (1994). The problem of assessing the biological role for a gene family of multifunctional calcium-and phospholipid-binding proteins. *Biochim. Biophys. Acta*, **1197**, 63–93.

SHIBATA S, SATO H AND MAKI M. (1992). Calphobindins (placental annexins) inhibit protein kinase C. *J. Biochem.*, **112**, 552–556.

SMITH PD AND MOSS SE. (1994). Structural evolution of the annexin supergene family. *Trends Genet.*, **10**, 241–246.

SMYTHE E, SMITH PD, JACOB SM, THEOBALD J AND MOSS SE. (1994). Endocytosis occurs independently of annexin VI in human A431 cells. *J. Cell Biol.*, **124**, 301–306.

THEOBALD J, SMITH PD, JACOB SM AND MOSS SE. (1994). Expression of annexin VI in A431 carcinoma cells suppresses proliferation: a possible role for annexin VI in cell growth regulation. *Biochim. Biophys. Acta*, **1223**, 383–390.